# Lymphocyte level and selected cognitive functions in patients with schizophrenia – preliminary results

**DOI:** 10.1192/j.eurpsy.2024.1591

**Published:** 2024-08-27

**Authors:** B. Nycz, K. Krysta

**Affiliations:** Department of Rehabilitation Psychiatry, Medical University of Silesia, Katowice, Poland

## Abstract

**Introduction:**

Schizophrenia is a mental disorder characterized by negative symptoms, such as cognitive impairment. Recent reports indicate the importance of the immune system in the pathophysiology of schizophrenia. The development of inflammation affects cognitive functioning.

**Objectives:**

The aim of the study was to analyze the association between the level of lymphocytes in venous blood and selected cognitive functions in patients with schizophrenia.

**Methods:**

Lymphocyte levels were determined in the venous blood of patients suffering from schizophrenia and the control group. Additionally, a verbal fluency test (VFT) and a Stroop test were conducted on the same day. The VFT evaluates the ability to express words, and the Stroop test assesses verbal working memory. The inclusion criteria were age up to fifty years, and for the study group – diagnosis of schizophrenia and treatment with neuroleptics. Exclusion criteria included organic brain diseases, electroconvulsive therapy, and use of benzodiazepines within 48 hours before the study. Currently, six patients and six healthy people have been studied.

**Results:**

Patients diagnosed with schizophrenia have an increased lymphocyte concentration in the blood compared to healthy individuals constituting the control group. There are discrepancies in the results of the phonemic fluency test, no significant differences were found between schizophrenics and the control group. Healthy men and women achieved higher results in the semantic fluency test compared to men and women with schizophrenia. Women constituting the control group achieved higher results in the Stroop test compared to women suffering from schizophrenia. Table 1 illustrates the concentration of lymphocytes in venous blood and the number of points in the phonemic fluency test, semantic fluency test, and in the Stroop test of the study and the control groups.

**Image:**

**Figure:**
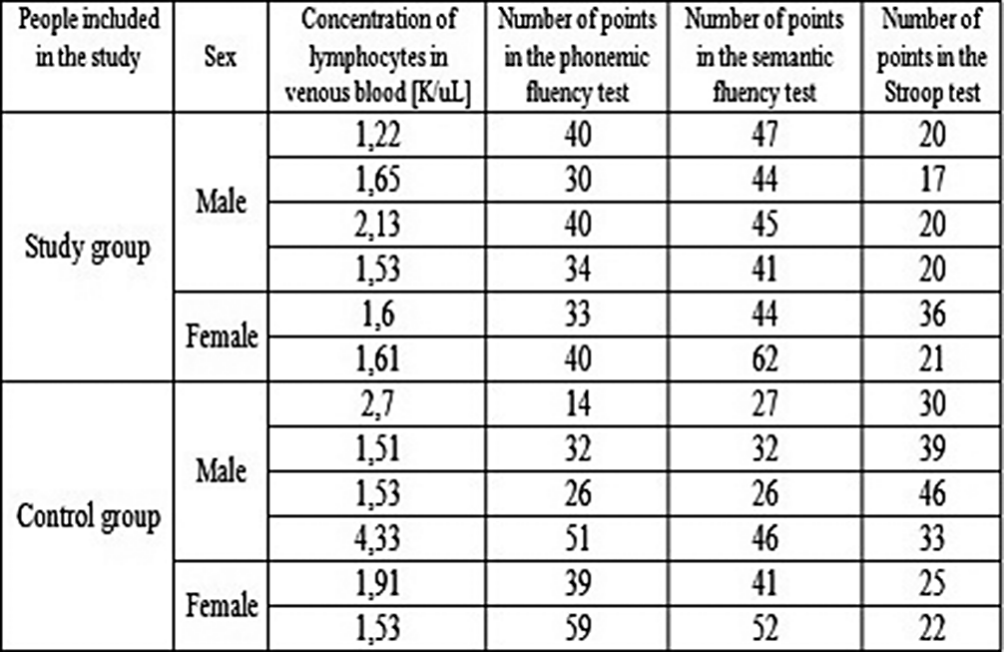

**Conclusions:**

Patients with schizophrenia are characterized by higher levels of immune system parameters and worse results in terms of semantic fluency. Men with schizophrenia showed no verbal working memory deficits. In turn, women with schizophrenia obtained worse results in the verbal working memory test. In conclusion, there is evidence of immune system activation in schizophrenia, which affects the cognitive functioning of patients.

**Disclosure of Interest:**

None Declared

